# Precision Medicine in Sepsis and Septic Shock

**DOI:** 10.3390/jcm11185332

**Published:** 2022-09-11

**Authors:** Alejandro Suarez-de-la-Rica, Emilio Maseda

**Affiliations:** 1 Department of Anesthesiology and Surgical Critical Care, Hospital Universitario Marqués de Valdecilla, 39008 Santander, Spain; 2 Department of Anesthesiology and Surgical Critical Care, Hospital Universitario La Paz, 28046 Madrid, Spain

Sepsis is defined as a potentially fatal organ dysfunction induced by a dysregulated host response to infection. New definitions of sepsis and septic shock were established in 2016. Systemic inflammatory response syndrome criteria were eliminated to identify sepsis. Sepsis-2 “severe sepsis” criteria and Sepsis-3 “sepsis” criteria were assimilated, and organ dysfunction grading was standardized by using the Sequential Organ Failure Assessment (SOFA) score. A quick SOFA (qSOFA) was introduced in the early detection of sepsis outside intensive care units, with a limited diagnostic accuracy [1].

The incidence of sepsis has increased, presumably due to the growing aging of the population; several studies have evidenced a relationship between age and incidence of sepsis and a higher number of people with disease comorbidities [2]. Data from high-income countries are mostly extrapolated from large retrospective database investigations, by identifying sepsis with ICD-coding strategies. Up to 49.9 million cases of sepsis were recorded globally in 2017 [3]. Sepsis-2 severe sepsis and Sepsis-3 sepsis had similar incidence rates in one analysis from the United Kingdom that was performed in critically ill adult patients [4].

Several studies demonstrated a lower mortality associated with sepsis over the years [5,6,7]. Even when Sepsis-2 severe sepsis and Sepsis-3 sepsis were compared, a similar hospital mortality was found with both definitions, with a similar decrease from 2011 to 2015 (33% to 30%) [4]. Possible reasons for this decrease in mortality are improvements in diagnostic procedures with quicker interventions, earlier and broader-spectrum antibiotic treatment, or more aggressive supportive therapy. Improved compliance with the resuscitation bundle of the Surviving Sepsis Campaign may have contributed to the decline in mortality as well [8].

However, the total number of patients that die as a result of sepsis is growing, amounting to more than 5 million deaths worldwide every year. These figures make sepsis a major public health concern [9]. Likewise, although the mortality of septic shock in patients included in randomized controlled trials decreased in recent years [10,11,12,13,14], in “real-life” patients, the mortality of septic shock patients according to the Sepsis-3 definition continues to be extremely high, as assessed by a recent retrospective study performed in more than 197,000 critically ill patients in the United Kingdom. This study found that hospital mortality for Sepsis-3 septic shock was 56% in 2015, without any decrease in the period of 2011–2015 [4]. This unacceptably high mortality has led to demands for new evidence to try to improve prognosis.

Several areas of uncertainty require further research. First, the absence of a reliable diagnostic marker to allow the early identification of sepsis is an issue. In this sense, a novel sepsis biomarker, the pancreatic stone protein, has been studied for identification of infection, and its diagnostic performance is better than most current biomarkers with a good prognostic value too [15].

Second, the lack of an adequate method to guide resuscitation presents an issue. Evidence that demonstrates the beneficial effects of advanced hemodynamic monitors or early goal directed therapy to accomplish specific targets is lacking [10,11,12].

Third is the issue of the unavailability of a treatment that changes the course of the disease. Most attempts to modify the inflammatory response have failed [13,14,16]. There are several potential reasons that may explain why most trials have not demonstrated any benefit in outcomes [17]. One potential reason may be the choice of an inadequate outcome, as short-term mortality has been the primary outcome in most trials. In recent trials, mortality rates of control groups were around 20%; therefore, demonstrating an improvement in outcomes is difficult.

Nevertheless, the factor probably most involved in the failure of trials is the heterogeneity of study populations. Sepsis is a syndrome difficult to define and characterize, produced by many different microorganisms in patients with variable age and comorbidities, and with different genetic susceptibility and immune responses.

Clinical phenotypes should be recognized in order to identify groups of patients that may respond to certain interventions. Seymour et al. defined four clinical phenotypes that showed adequate correlation with host-response biomarkers and clinical outcomes [18]. Hemodynamic phenotyping with echocardiographic and clinical parameters has also been proposed [19]. In the ANDROMEDA-SHOCK-2 trial, resuscitation based on clinical and hemodynamic phenotypes will be tested to find a possible beneficial effect in mortality [20]. These different phenotypes might be integrated in daily practice to implement precision medicine.

The use of biomarkers related to the proposed intervention is another method by which to improve homogeneity. Since patients with sepsis have a different underlying pathophysiology despite similar clinical presentations, biomarkers may play an important role in precision medicine. For example, Panacek et al. found that anti-tumor necrosis factor (TNF) reduced mortality rates in patients with increased interleukin-6 levels [21]. Immunological dysfunction is a major pathogenic event in many patients with sepsis, and understanding it may lead to future treatments with immuno-modulatory drugs [22]. Although this approach remains experimental at present, metabolomics is defined as the study of physiologically relevant small molecules that are responsible for metabolic processes in organisms and can be used as direct biomarkers of biochemical activity, with a more direct correlation with phenotypes [23].

In conclusion, sepsis and septic shock require a personalized approach to try to improve prognosis. In this Special Issue, contributions with quality evidence regarding all aspects of sepsis and septic shock are welcome, in order to improve the management of this complex syndrome.
